# Effect of tear collection on lacrimal total protein content in dogs and cats: a comparison between Schirmer strips and ophthalmic sponges

**DOI:** 10.1186/s12917-018-1390-7

**Published:** 2018-03-01

**Authors:** Lionel Sebbag, Emily M. McDowell, Patrick M. Hepner, Jonathan P. Mochel

**Affiliations:** 1 Department of Veterinary Clinical Sciences, Iowa State University, College of Veterinary Medicine, Ames, IA 50011 USA; 2 Lloyd Veterinary Medical Center, Iowa State University, College of Veterinary Medicine, Ames, IA 50011 USA; 3 Department of Biomedical sciences, Iowa State University, College of Veterinary Medicine, Ames, IA 50011 USA

**Keywords:** Total protein content, Tear fluid, Tear flow, Schirmer tear test, Ophthalmic sponge

## Abstract

**Background:**

Quantification of lacrimal total protein content (TPC) is an important tool for clinical scientists to understand disease pathogenesis, identify potential biomarkers and assess response to therapy, among other applications. However, TPC is not only affected by disease state but also by the method used for tear collection. Thus, the purpose of this study is to determine the impact on TPC of two methods of tear collection in dogs and cats: Schirmer strips and polyvinyl acetal (PVA) sponges.

**Methods:**

(i) In vivo - Ten healthy dogs and 10 healthy cats were examined. Each animal underwent two sessions, separated by 10 min, in which a Schirmer strip was placed in one randomly selected eye until the 20-mm mark was reached, while a strip of PVA sponge was placed in the other eye for 1 min. (ii) In vitro - Schirmer strips and PVA sponges were spiked with various volumes of four bovine serum albumin solutions (0.5, 4, 10, and 20 mg/mL).

In both experiments, the wetted absorbent materials were centrifuged for 1 min, and the TPC was quantified on the extracted fluid using Direct Detect™ infrared spectroscopy.

**Results:**

Lacrimal TPC in dogs and cats ranged from 5.2 to 14.6 mg/mL and from 6.2 to 20.6 mg/mL, respectively. In cats, TPC was significantly lower with Schirmer strips vs. PVA sponges (*P* < 0.001). In dogs, the volume absorbed by PVA sponges was negatively correlated with TPC (*r* = − 0.48, *P* = 0.033). The inter-session coefficient of variation was significantly lower with Schirmer strips vs. PVA sponges in both species (*P* ≤ 0.010). In vitro, both absorbent materials resulted in a ‘concentrating effect’ of the TPC obtained post-centrifugation, which was most pronounced when the volume absorbed was low, especially for Schirmer strips.

**Conclusion:**

Schirmer strips provide a repeatable method to quantify lacrimal TPC in dogs and cats, although care should be taken to absorb sufficient volumes of tears to minimize the concentrating effect from the absorbent material.

## Background

Tears are a complex biological fluid composed of a mixture of substances. Proteins, one of the major classes of compounds in tear fluid, promote ocular surface health through various functions that include anti-microbial defense, anti-oxidation and modulation of wound healing [1]. Recent developments in proteomics have identified over 1500 proteins in the tear fluid [2], some being secreted by the lacrimal glands (‘lacrimal-derived’, e.g. lysozyme, lactoferrin, lipocalin and IgA) and some leaking into tears from the conjunctival capillaries (‘serum-derived’, e.g. albumin, transferring and IgG). Changes in tear protein composition have been closely associated with disease state, not only for eye-specific conditions such as dry eye [3] and allergic conjunctivitis [4], but also for systemic disorders such as diabetes mellitus [5] and cancer [6]. For instance, the expression pattern of proteins in tears is different in dogs with various cancers when compared to healthy subjects [6]. Thus, analysis of tear protein can help identify new biomarkers, better understand disease pathogenesis, and develop new diagnostic tools and therapeutics. However, it is often challenging to draw accurate conclusions and make cross-study comparisons because the protein content in tears is not only affected by disease state, but also largely by the method of tear collection used by each investigator.

The collection technique can strongly influence the protein profile in a tear sample. For instance, a higher total protein content (TPC) was found in tear samples obtained with Schirmer strips when compared to capillary tubes in humans [7] and dogs [8], largely due to a greater amount of serum-derived proteins, although these findings were not confirmed in a recent study by Posa and colleagues [9]. Direct sampling of tear fluid with capillary tubes, albeit commonly used in humans [7, 9], presents several drawbacks that limit its use in veterinary species. In particular, the collection process with capillary tubes is slow and the volume obtained is generally small, with a reported average collection time of 6–7 min to obtain ≤ 10 μL of non-stimulated tear fluid in cats [10]. Combined with the uncooperative nature of most veterinary patients, these limitations make it challenging to avoid reflex tearing or accidental trauma to the eye in a reliable and repeatable manner. The challenges of using capillary tubes also exist in human subjects, especially in individuals with reduced tear volume [11]. Thus, some investigators have encouraged the use of absorbent materials as an alternative method for tear collection: besides being safe, absorbent materials likely provide an enriched sample by retaining compounds present on the ocular surface [11]. For instance, Li and colleagues showed that defensins, key antimicrobial peptides expressed by corneo-conjunctival epithelia [12], are upregulated in Schirmer-collected tear samples of patients with Sjögren’s-related dry eye [13].

The purpose of our study is to evaluate the effect of tear collection with two different absorbent materials on TPC in dogs and cats, namely Schirmer strips and polyvinyl acetal (PVA) ophthalmic sponges. The study design combines both in vivo and in vitro experiments *–* the former aims to capture the impact of normal ocular surface physiology (e.g. tear flow, reflex tearing) on the parameter evaluated, while the latter aims to determine the impact of various protein concentrations on the adsorptive properties of the absorbent materials, i.e. the amount of protein retained by the Schirmer strips and PVA sponge after tear extraction.

## Methods

### In vivo evaluation

#### Animals

Ten dogs and 10 cats were enrolled in the study. After obtaining informed consent from owners, all subjects were confirmed to be ophthalmoscopically healthy by slit-lamp examination,^1^ indirect funduscopy,^2^ tonometry,^3^ Schirmer tear test,^4^ and fluorescein staining. The canine population was comprised of two Labrador Retrievers, two Yorkshire terriers, and one dog of the following breeds: Greyhound, Great Pyrenees, Border Collie, English Bulldog, Poodle, and Chihuahua. The mean ± standard deviation (minimum-maximum) age and body weight of dogs were 7.7 ± 3.9 years (2–14 years) and 16.8 ± 8.7 kg (5.2–30.2 kg), respectively. The feline population was comprised of six Domestic Short Hair, two Domestic Long Hair, one Burmese and one Persian cat. The mean ± standard deviation (minimum-maximum) age and body weight of cats were 7.6 ± 3.9 years (3–15 years) and 4.9 ± 1.3 kg (3.1–6.8 kg), respectively. In both species, five subjects were castrated males and five were spayed females. The study was approved by the Institutional Animal Care and Use Committee of Iowa State University.

#### Tear collection

In each animal, the bent tip of a Schirmer strip^4^ was placed in the ventral conjunctival fornix of one randomly selected eye (coin toss), while a 4 × 10 mm strip of PVA ophthalmic sponge^5^ was placed in the ventral conjunctival fornix of the other eye, as previously described [14]. The Schirmer strip was removed when the 20-mm mark of wetness was reached, while the PVA sponge was removed after 60 s. The wetted Schirmer strip and PVA sponge were removed and placed in separate 0.2-mL Eppendorf tubes that were previously punctured at their bottom with a 18-gauge needle. The combinations were sealed into separate 1.5-mL Eppendorf tubes with adhesive tape and subsequently centrifuged at 3884 g for 1 min to extract the tear fluid.^6^ Tear collection and extraction were repeated 10 min later using the same randomized eye for each absorbent material. The volume of tears absorbed (VA) and recovered in each sample were calculated by the difference of post- and pre-collection weight^7^ of the 0.2-mL tubes, and the difference of post- and pre-centrifugation weight of the 1.5-mL tubes, respectively [14].

#### Total protein quantification

TPC was quantified in each sample using the Direct Detect™ infrared spectrometer.^8^ An aliquot of 2 μL of each sample was spotted on the membrane card and analyzed using phosphate-buffered saline (PBS)^9^ as blank. The card was inserted into the instrument and results were recorded in mg/mL using the included software. Since the linear range of the Direct Detect™ is 0.2–5.0 mg/mL, samples that resulted in a protein content greater than 5 mg/mL were diluted with PBS and re-analyzed. Total protein content was recorded in mg/mL, adjusting for any dilution when necessary.

### In vitro evaluation

Four protein concentrations (0.5, 4, 10 and 20 mg/mL) were evaluated, representative of the range of TPC obtained in vivo in canine and feline tears (in vivo part of the present study). Solutions of 0.5 mg/mL^10^ and 20 mg/mL^11^ bovine serum albumin (BSA) were obtained, and the latter was used to make solutions of 4 and 10 mg/mL BSA by 5- and 2-fold dilution with PBS, respectively.

Three volumes of each BSA solution were spiked onto different sets of Schirmer strips and PVA sponges. The volumes selected were representative of the range of volume absorbed by PVA sponges from the present in vivo experiment and from a previous publication [14], while the range of volume absorbed by Schirmer strips from another experiment (unpublished data) in which Schirmer tear test values varied from 1 to 35 mm/min. This is because the present study design (i.e. standardized tear collection until the 20-mm mark was reached) had limited the range of volume absorbed by Schirmer strips.

The three volumes of BSA solutions were defined as:

- “Low volume”: Representing the 10th percentile of tear volume absorbed in vivo, which is 10 μL for both absorbent materials;
- “Intermediate volume”: Representing the 50th percentile of volume absorbed in vivo, which is 20 μL for STT and 25 μL for PVA sponges;
- “High volume”: Representing the 90th percentile of volume absorbed in vivo, which is 30 μL for STT and 60 μL for PVA sponges.

In summary, 12 combinations were evaluated in vitro for both Schirmer strips and PVA sponges: low, intermediate and high volumes of 0.5, 4, 10, and 20 mg/mL BSA solutions.

First, each of the four BSA solutions was analyzed 10 times for its total protein content using the Direct Detect™ infrared spectrometer, and results (expressed in mg/mL) were used as ‘control’ for comparisons with the experimental groups. Second, each of the 12 combinations was evaluated by spotting the set volume of BSA solution in 10 separate Schirmer strips and 10 separate PVA sponges. The wetted Schirmer strips and PVA sponges were subsequently handled in a similar fashion than the in vivo study, and the TPC of fluids extracted by centrifugation was evaluated with the Direct Detect™ infrared spectrometer.

### Data analysis

Normality of the data was assessed with the Shapiro–Wilk test. In each species, the Wilcoxon signed-rank test was used to compare the TPC obtained in vivo by Schirmer strips and PVA sponges, as well as the coefficients of variation (CV%) between both tear collection sessions. Associations between TPC, volume of tears absorbed, age and body weight were examined by the Spearman’s rank correlation test for each species and each absorbent material. The Mann-Whitney test was used to assess differences between the TPC of each in vitro combination (volume/protein concentration) and the TPC of the corresponding BSA solution. Statistical analysis was performed using SigmaPlot version 13.0^12^, and values *P* < 0.05 were considered statistically significant.

## Results

### In vivo evaluation

**Fig. 1:**
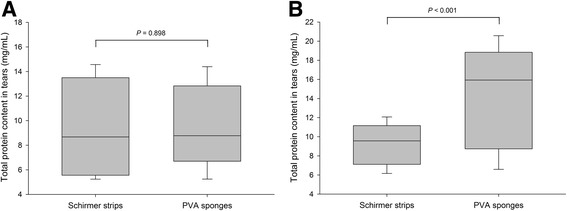
Box-and-whisker plots depicting the total protein content in tear samples collected in 10 healthy dogs (**a**) and 10 healthy cats (**b**) with either Schirmer strips or PVA sponges. Median values are shown by a horizontal line. First and third quartiles (25th and 75th percentiles) are represented by the lower and upper limits of the box, respectively. The 2.5th and the 97.5th percentiles are shown as the lower and upper whiskers, respectively

Data were not normally distributed for any parameter evaluated (*P* < 0.05), so results are presented as median and 95% range (2.5–97.5th percentiles). Median (95% range) canine TPC in tears was 8.7 mg/mL (5.2–14.6 mg/mL) with Schirmer strips and 8.8 mg/mL (5.2–14.4 mg/mL) with PVA sponges, a difference that was not statistically significant (*P* = 0.898; Fig. 1a). Median (95% range) feline TPC in tears was 9.6 mg/mL (6.2–12.1 mg/mL) with Schirmer strips and 15.9 mg/mL (6.6–20.6 mg/mL) with PVA sponges, a difference that was statistically significant (*P* < 0.001, Fig. 1b).

Inter-session comparison of TPC (initial vs. tear sample collected 10 min later) showed that the median (95% range) CV% was significantly lower (*P* = 0.020) in Schirmer strips (3.9%, 0.5–10.8%) vs. PVA sponges (10.6%, 2.2–75.0%) in canine patients. Similarly, the median (95% range) CV% was significantly lower (*P* = 0.010) in Schirmer strips (5.9%, 1.2–11.7%) vs. PVA sponges (23.7%, 0.4–57.3%) in cats.

**Fig. 2:**
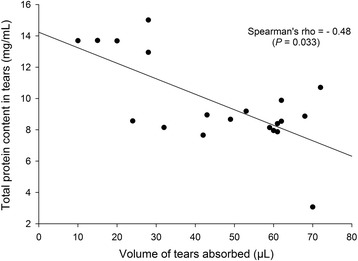
Spearman’s rank correlation testing for TPC and VA of tear samples collecting with PVA sponges in 10 healthy dogs. A moderate negative correlation was found (*Spearman’s rho* = − 0.48, *P* = 0.033)

A significant moderate negative correlation was noted between the volume of tears absorbed by the PVA sponges and the TPC quantified in tears of dogs (*Spearman’s rho* = − 0.48, *P* = 0.033; Fig. 2), but not in cats (*P* = 0.21). No correlations were noted between the volume of tears absorbed by Schirmer strips and TPC in either species (*P* ≥ 0.35). Similarly, no correlations were noted between age, body weight and TPC in either species (*P* ≥ 0.097).

### In vitro evaluation

**Fig. 3:**
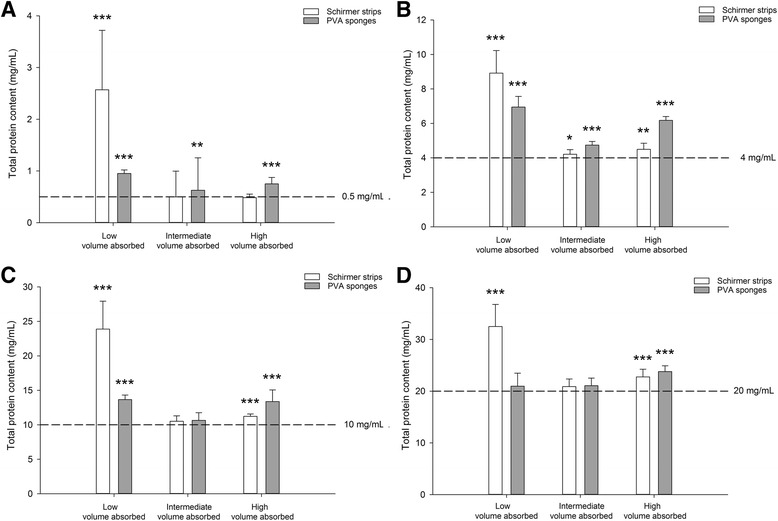
Bar charts depicting the mean + standard deviation of TPC from in vitro experiments evaluating 12 combinations of volume absorbed/protein concentration in both Schirmer strips (white bars) and PVA sponges (gray bars): low, intermediate and high volumes of 0.5 mg/mL (**a**), 4 mg/mL (**b**), 10 mg/mL (**c**), and 20 mg/mL (**d**) BSA solutions. In each panel, statistical differences between the absorbent materials and the control BSA solution are shown by asterisks: * *P* < 0.05, ** *P* < 0.01, *** *P* < 0.001

Results of the in vitro experiments are summarized in Fig. 3. Each figure panel describes a different concentration of BSA (0.5, 4, 10, and 20 mg/mL), representative of the range of TPC noted in the in vivo experiments.

The fluid extracted post-centrifugation often contained a significantly higher TPC (*P* ≤ 0.045) when compared to the standard BSA solution used to wet the Schirmer strips and PVA sponges. This ‘concentrating effect’ of the absorbent materials was variable, and depended upon two parameters (i) the volume of fluid absorbed, and (ii) the protein concentration of the solution.

Indeed, the ‘concentrating effect’ was most pronounced when the volume absorbed was low, especially for Schirmer strips. For instance, when spiking 10 μL (‘low volume’) of 4 mg/mL BSA solution on Schirmer strip and PVA sponges (Fig. 3b), the recorded mean TPC of the fluid extracted post-centrifugation was 8.9 mg/mL and 6.9 mg/mL, respectively. These values represent a significant increase of TPC by 222% and 172% compared to the control BSA solution of 4 mg/mL, respectively (*P* < 0.001).

To a lesser degree, the original concentration of proteins also influenced the resulting TPC quantified in the fluid obtained post-centrifugation. For instance, when a similar ‘intermediate’ volume of 25 μL was spiked onto PVA sponges, the recorded mean TPC of the extracted fluid did not vary significantly from the control BSA solutions for 10 and 20 mg/mL (*P* > 0.05), but was significantly greater for 0.5 mg/mL (*P* = 0.003) and 4 mg/mL (*P* < 0.001).

## Discussion

Tears collected with an absorbent material and extracted with centrifugation yielded a TPC of 5.2–14.6 mg/mL in dogs and 6.2–20.6 mg/mL in cats. These values are likely accurate estimates of the ‘true’ TPC for samples in which the tear volume absorbed was around 20–25 μL (i.e. median VA in vivo), but may represent an over-estimation of TPC for samples with low VA and, to a lesser degree, those with high VA. Indeed, our in vitro data showed a clear ‘concentrating effect’ from both absorbent materials when VA was low (10 μL), which was noticeable across the entire range of protein levels tested and more pronounced with Schirmer strips than PVA sponges. Despite centrifugation, our group has previously shown that a relatively large portion of fluid was retained by ophthalmic sponges when the volume of tears absorbed was low [14], especially for cellulose-based sponges. That explains the greater concentrating effect noted in the present study for Schirmer strips, which are hydrophilic materials manufactured from cellulose filter paper [15].

Regardless, the level of proteins reported herein is superior to the one described in tear fluid collected with capillary tubes in dogs (2.6 mg/mL) [16] and cats (10.4 mg/mL) [10]. Consistent with older reports in human subjects, the higher TPC in absorbent materials such as Schirmer strips vs. capillary tubes is presumably related to an increased in serum-derived proteins due to conjunctival irritation [7, 17]. However, these findings are not consistent across the scientific literature as recent studies have described an equal [9] or inferior [18] amount of proteins in Schirmer-collected tears when compared to capillary tubes. Thus, it is likely inaccurate to compare the findings of various studies based on the tear collection method alone. Rather, one should also consider how the tears were extracted from the absorbent material - i.e. centrifugal force [9], elution with a solvent [8, 15], or a combination of both [18, 19] - as well as the method used by the investigator to quantify the protein content.

In the present study, the assay used to quantify TPC relies on infrared spectrometry that measures the amide bond absorbance across all proteins and peptides contained in the sample. This method improves the accuracy of the assay as compared to colorimetric methods [20]. Although extensively used for TPC in tear fluid because of their simplicity and sensitivity [3, 8, 16], colorimetric methods such as Lowry and Bradford have drawbacks that can result in under- or over-estimation of TPC in tears [21]. For instance, the Bradford assay highly depends on the specific protein composition in a sample [22], and this becomes a considerable disadvantage when evaluating TPC in a complex protein mixture such as tear fluid. Further, since serum albumin is not a major component of tear proteins [23, 24], using BSA as a standard likely affects the TPC obtained by the colorimetric assay; in fact, TPC in tear fluid significantly differs whether BSA or IgG is used as the standard for the method [21].

A significant negative correlation was noted between tear flow rate and TPC in dogs, consistent with findings by Fullard and colleagues [3]. In another canine study, individuals with epiphora consistently had low tear fluid protein levels while subjects with dry eye had a high protein concentration [23]. In contrast, there was no correlation between tear flow rate and TPC in our feline subjects, and a recent publication in human patients showed a positive correlation between TPC and the Schirmer readings [19]. The reason for these discrepancies remains unclear and warrants further investigation. Further, body weight and age were not correlated with TPC in either species, despite the reported positive correlation between body weight and tear volume absorbed in dogs [14]. This finding, as well as potential impact of breed on TPC, should be verified in a larger population size.

Importantly, our study found that the repeatability of protein quantification in tears was greater with Schirmer strips when compared to PVA sponges, as determined by significantly lower inter-session CV% in both species. This could be explained by the lower variability in tear volume absorbed by Schirmer strips. Indeed, the mm- marks of Schirmer strips can be used to standardize the volume of tears absorbed at each collection. In the present study, the Schirmer strips were left in the conjunctival fornices of dogs and cats until the 20-mm mark was wetted, thus reducing the variability in VA between sessions and therefore improving the repeatability of TPC measurement. On the other hand, standardizing the duration of PVA-collection to 60 s did not reduce the variability in VA obtained between sessions; for instance, the VA in one representative dog was 20 μL at the initial sampling, and 43 μL when tear collection was repeated 10 min later. Although not evaluated in the present study, the extraction of tears from Schirmer strips could be optimized by ‘washing’ the strip with a solvent to help retrieve any small amount of protein that remained on the absorbent material post-centrifugation [9].

The present study was limited to describing total protein content, i.e. ‘gross’ protein quantification in the tear fluid. Further studies could investigate which specific tear proteins (e.g. albumin, lactoferrin) are most affected by changes in tear flow rate, or by the adsorptive properties of the Schirmer strips and PVA sponges used for tear collection. A recent study in humans determined that proteins are preferentially retained by Schirmer strips based on physicochemical factors such as molecular weight and surface charge [15].

Nevertheless, results of the present study are valuable given the potential applications of total protein quantification in veterinary medicine. In a clinical setting, TPC can be used to better characterize various ocular surface diseases and assess their response to therapy, as exemplified by keratoconjunctivitis sicca in dogs [23] and corneal sequestrum in cats [25]. In a research setting, TPC can be used to standardize the amount of tear sample to be used for assessing inflammatory mediators [19], in-depth proteomics [26] and others.

## Conclusion

Schirmer strips are more reliable than PVA sponges (i.e. lower inter-session CV%) for quantification of TPC in canine and feline tears. However, care should be taken to absorb sufficient volumes of tears with Schirmer strips to minimize the concentrating effect from the absorbent material.

## Footnotes

1 SL-17; Kowa Company, Ltd., Tokyo, Japan

2 Keeler Vantage; Keeler Instruments, Inc., Broomall, PA, USA

3 TonoVet; Icare Finland Oy, Espoo, Finland

4 Eye Care Product Manufacturing, LLC, Tucson, AZ, USA

5 Ultracell^®^ Eye Wick, Beaver-Visitec International, Inc., Waltham, MA, USA

6 VWR® Mini Centrifuge, VWR International, Radnor, PA, USA

7 Gemini-20, American Weight Scales Inc., Norcross, GA, USA

8 Millipore-Sigma, St Louis, MO, USA

9 Gibco™, Thermo Fisher Scientific, Inc., Waltham WA, USA

10 Bovine Serum Albumin, VWR International, Radnor, PA, USA

11 Bovine Serum Albumin, Fischer Scientific, Hampton, NH, USA

12 Systat Software Inc., San Jose, CA, USA
